# Building Resilience Against ViolencE (BRAVE): protocol of a parenting intervention for mothers and fathers with post-traumatic stress disorder in Pakistan

**DOI:** 10.1017/gmh.2021.42

**Published:** 2022-03-17

**Authors:** Nasim Chaudhry, Sana Farooque, Tayyeba Kiran, Ozlem Eylem-van Bergeijk, Imran B. Chaudhry, Rakhshi Memon, Mina Husain, Panoraia Andriopoulou, Mowadat Hussain Rana, Farooq Naeem, Nusrat Husain

**Affiliations:** 1 Pakistan Institute of Living and Learning (PILL), Karachi, Pakistan; 2 Centre for Psychiatry, Queen Mary University of London, London, UK; 3 Division of Psychology and Mental Health, The University of Manchester, Manchester, UK; 4 Department of Psychiatry, Ziauddin University and Hospital, Karachi, Pakistan; 5 The Manchester Global Foundation (MGF), Manchester, UK; 6 South London and Maudsley NHS Foundation Trust, London, UK; 7 Department of Psychology, Manchester Metropolitan University, Manchester, UK; 8 Department of Psychiatry, Toronto University, Toronto, Canada; 9 Lancashire & South Cumbria NHS Foundation Trust, Lancashire, UK

**Keywords:** Learning through play, parenting intervention, post-traumatic stress, trauma-focused CBT, Trauma

## Abstract

**Background:**

Prevalence of post-traumatic stress disorder (PTSD) is high in Pakistan both due to natural disasters and ongoing conflicts. Offspring of trauma survivors are at increased risk for mental and physical illnesses. Parental PTSD has been linked to troubled parent–child relationships, behaviour problems, trauma symptoms, and depression in children. This study aims to explore the acceptability, feasibility and indications of the effectiveness of group learning through play plus trauma-focused cognitive behaviour therapy (LTP Plus TF-CBT) for parents experiencing PTSD.

**Methods/Design:**

This is a two-arm pilot cluster randomised controlled trial (RCT). We aim to recruit 300 parents with a diagnosis of PTSD. The screening will be done using the Impact of Event Scale-Revised. Diagnosis of PTSD will be confirmed using the Clinician-Administered PTSD Scale-5 (CAPS-5). Union Councils from Peshawar and Karachi will be randomised into either group LTP Plus TF CBT arm or treatment as usual (TAU). The intervention includes 12 sessions of LTP Plus TF-CBT delivered weekly in the first 2 months and then fortnightly in a group setting by trained psychologists. The groups will be co-facilitated by the community health workers (CHWs). Parents will be assessed at baseline and 4th month (end of the intervention), using the Patient Health Questionnaire (PHQ-9), Generalised Anxiety Disorder (GAD-7) Scale, Client Service Receipt Inventory (CSRI), and Ages and Stages Questionnaire (ASQ-3)

**Discussion:**

This trial would help build an understanding of the acceptability, feasibility and indications of the effectiveness of a low-cost parenting intervention.

## Background

The American Psychological Association has defined trauma as an ‘emotional response to a terrible event such as an accident, natural disaster or rape etc’ (APA, 2016). Exposure to trauma leads to post-traumatic stress disorder (PTSD) (Stein *et al*., 2016). Low and middle-income countries (LMICs) have higher trauma exposure, with high rates of PTSD in post-conflict areas (Atwoli *et al*., 2015). Post-trauma symptoms can particularly overburden parents and cause withdrawal and conflict among family members (Harrison *et al*., 2014). It has been reported that trauma may be transmitted to offspring via parenting practices burdened with severe parental emotional distress (Song *et al*., 2014). Hence, trauma and PTSD in parents leads to increased risk for mental and physical illnesses in children (Creech and Misca, 2017; Palosaari *et al*., 2013), behavioural issues along with trauma-related symptoms (Creech and Misca, 2017), disturbed parent–child relationships such as child abuse (Cross *et al*., 2018; Montgomery *et al*., 2019), and high incidence and intergenerational spread of family violence (Olema *et al*., 2014; Timshel *et al*., 2017).

The prevalence of PTSD in Pakistan both due to natural disasters and on-going conflict is high (Khan *et al*., 2015). In a survey study (*N* = 500) in Islamabad in Pakistan, it was found that 20.2% people reported that they experienced direct trauma during the last 12 months (Naeem *et al*., 2012). Additionally, nearly half of those (45.3%) who directly experienced trauma in addition to an indirect exposure scored positive for PTSD, compared with one-fifth (20.8%) of those who only experienced indirect trauma (Naeem *et al*., 2012). Pakistan has been in constant turmoil for decades; the threat level varies from city to city and locality to locality. The incident on the 16th December (2014) at the Army Public School (APS) in Peshawar, Khyber Pakhtunkhwa (KPK) province, which involved the shooting of young children, teachers and other staff in addition to an attack a year later at Bacha Khan University (in KPK) in January 2016, have had a major impact locally, nationally and internationally. A further terrorist attack on students at Peshawar University on 1st December 2017 again left the whole country in a state of shock and emotional turmoil. There is evidence that such events affect even those who hear about them but certain groups, such as the parents of school-going children are most affected (Khalily, 2011).

A healthy and positive development is consistent with positive,nurturing and secure parent-child attachment, family-community, and community-society relationships that reinforce self-worth, minimise frustration, and promote self-confidence (Winston and Chicot, 2016). Positive and nurturing parent-child relationships are largely dependent upon parents' ability to offer a secure and consistent environment for their children. It is reported that parental involvement, in the form of family routines, parental monitoring and supportiveness, is a protective factor for children facing conflict and disruption (Dubow *et al*., 2012).

The impact of early parenting interventions on parents and infants has been reviewed and meta-analysis reported that parenting interventions are effective in improving parenting responsiveness (*d* = 0.77) (Mihelic *et al*., 2017). An integrated intervention called learning through play plus cognitive behaviour therapy (LTP Plus TF-CBT) has found to be effective in low resource settings such as Pakistan (Rahman *et al*., 2009; Husain *et al*., 2017a, 2020). The LTP aims to stimulate early child development and its central feature is a pictorial calendar devised for parents, depicting eight successive stages of child development from birth to 3 years (Centre, 2002). Evidence also exist on parenting interventions delivered in the face of conflict and displacement in Syria (El-Khani *et al*., 2016).

Primary findings indicate that caregivers who have difficulties managing their own stress in addition to the stress caused by the child's behavioural and emotional changes require support maintaining positive parenting strategies. Trauma-focused cognitive behaviour therapies (TF-CBTs) are defined as interventions that include a systematic focus on processing trauma memories (Ennis *et al*., 2020) and can be a potential intervention for trauma survivor parents. Trauma-focused cognitive-behavioural therapy has proven very helpful and is often the treatments of choice for individuals with PTSD (Watkins *et al*., 2018). However, implementation of trauma-focused interventions is hindered in LMICs because of limited access to evidence-based psychological treatments and socio-political instability (Chen *et al*., 2017).

We propose to investigate the acceptability, feasibility and preliminary effectiveness of a manual assisted learning through play (LTP) plus culturally adapted TF-CBT among parents with young children experiencing PTSD. LTP Plus is proposed as a low-cost intervention to improve parents' mental and physical health and promote healthy child development. The activities in LTP are meant to enhance the social and emotional development of children. The activities are designed in a way which requires parents to be sensitive to the children's needs and actively engage with them, thus strengthening attachment (Husain *et al*., 2017b, 2020). In the proposed study LTP will be integrated with adapted trauma-focused CBT (Naeem *et al*., 2015; Latif *et al*., 2020) and will be delivered in a group format. TF CBT delivered in group settings uses cognitive restructuring techniques to facilitate each group member in processing their trauma experience. Each participant in a group has the opportunity to narrate his or her personal story as other group members listen. Hence, there is potential for all group members to participate in trauma processing through both recounting their own traumatic experience as well as vicarious experiences of others'. TF CBT group models encourage: strength of the personal narrative, power of group support, members ‘stand together’ and hear the experiences of others without judgment (Foy, Eriksson, and Trice, 2001).

## Research question

Whether the LTP Plus TF CBT is more feasible, acceptable and potentially effective as compared to the routine care for parents of young children in Pakistan?

### Hypothesis

The LTP Plus TF CBT will be more feasible, acceptable and potentially effective as compared to the routine care for parents of young children in Pakistan.

## Method

### Design

This is a two-arm pilot cluster RCT. Both qualitative and quantitative assessments will be administered to all eligible participants at baseline and after 4 months (end of the intervention).

### Randomisation

Randomisation will be computer-generated by an off-site statistician. The unit of a cluster will be Union Councils. A union council forms the second-tier of local government and fifth administrative division in Pakistan, The Union Councils will be randomly allocated to one of the two arms; arm-1 is the intervention group receiving LTP Plus and TF-CBT whereas Arm-2 is routine care.

### Blinding

The proposed study will be a single-blind study. The outcome assessors will be blind to the allocation status.

### Study site and population

Participants will be recruited from community settings (e.g. primary care) in Peshawar and Karachi with the assistance of community health workers (CHWs). Karachi is the capital of the province of Sindh, and the largest city in Pakistan, the world's third most populated city (Karachi Metropolitan Corporation). Karachi is a metropolitan city with diverse populations of political and economic migrants, refugees from different religious, linguistic, provincial and national origins who move to settle permanently. Peshawar is the capital of the province KPK, situated close to the border of Afghanistan. It has gone through major changes as a result of the Soviet-Afghan war and ongoing conflicts.

The parents with children aged between 3 and 6 years old will be screened with the Impact of Event Scale-Revised (IES-R) 22-item scale (Sundin and Horowitz, 2002). Those scoring 24 or above will be eligible for a full assessment of PTSD measured by Clinician-Administered PTSD Scale-5 (CAPS-5). The IES-R has been successfully used in Pakistan (Latif *et al*., 2020).

#### Inclusion criteria

- Parents over the age of 16 years
- Living with their children age 3–6 years
- Who meet the criteria for CAPS-5
- Able to give informed consent

#### Exclusion criteria

- Parents with diagnosed severe physical or learning disability or severe mental illness which prevents them from attending the LTP Plus group intervention. This will be assessed by the research team at the screening stage. Any disabilities will be identified by a relevant clinician (e.g. Psychiatrist).
- Those who expressed active suicidal ideation during screening.
- Current use of anti-depressant medication reported by the parents.

### Procedure

Trained CHWs will approach potential participants in community settings (e.g. primary care, basic health units) in both intervention and control clusters. CHWs will assess potential participants against eligibility criteria (accept the diagnosis of PTSD using CASP). All potentially eligible participants will be invited for a meeting with a trained researcher who will then administer CASP on otherwise eligible participants. After screening, a detailed Participant Information Leaflet (PIL) will be provided to eligible parents and written (thumb impression for participants who are unable to read and write) consent will be obtained. Trained researchers will complete baseline assessment with all consented participants. Participants in the intervention cluster will receive 12 group sessions of LTP Plus TF CBT over the period of 4 months. A follow-up assessment will be done with all the participants 4 months after baseline.

### Intervention

#### Learning through play + trauma-focused cognitive behaviour therapy (LTP + TFCBT)

The culturally adapted parent-focused group intervention (LTP plus TF-CBT) will be delivered to parents by masters-level trained psychologists supported by CHWs weekly during the first 2 months and then fortnightly at schools either at school drop-off or pick-up time depending on participant's preference and other community settings. The parenting component of the intervention (LTP) will be led by the CHWs and psychologists will lead the TF CBT component of the intervention. We have published a large cluster RCT of integrated parenting intervention called LTP Plus CBT for depressed mothers where CHWs successfully delivered intervention facilitated by the psychologists (Husain *et al*., 2021). The Intervention will be delivered in 12 sessions of 60–90 min in addition to routine care. The LTP plus TF-CBT has two components;

1. Culturally adapted learning through play (LTP)
2. Culturally adapted trauma-focused cognitive behaviour therapy (TF-CBT)

LTP aims to enable parents to improve their child's psychical and psychosocial development by educating about child development and the importance of parent-child play. The pictorial calendar, which starts from birth and exhibits eight stages of healthy child development until 6 years of age, is an important feature of LTP. To enable a better understanding of how learning and attachment works, the calendar comes with culturally adapted illustrations of parent-child play (Husain *et al*., 2020).

Component 2 of this integrated group intervention involves a culturally adapted manual-assisted cognitive behaviour therapy (CBT) for PTSD, based on the cognitive model by Ehlers and colleagues (Ehlers *et al*., 2005). The cognitive appraisals, memory characteristics and behavioural strategies which maintain PTSD are all considered while developing this model. The aim is to encourage careful questioning to modify negative appraisals of the trauma. The culturally adapted manual BASID (Baad Az Sadma Zehni Dabao) Ki Kahani – The Story of Basid (Latif *et al*., 2020), consists of nine chapters and in addition to psycho-education and culturally sensitive advice on improving relationships, it includes practical exercises on; dealing with avoidance, behavioural activation, problem-solving and dealing with unhelpful ways of thinking through thought restructuring. The manual uses culturally sensitive idioms of distress, stories, and expressions. For example, the principle of exposure is explained through Aesops' famous fable about a lion who fears a lion in the water when he tried to drink water from a pond. It is only when he jumps over the lion that the lion disappears. The manual describes stories of three persons who have experienced trauma, and how they managed their traumatic stress.

Chapters of BASID are woven throughout the intervention curriculum, as evidenced from table 3, each session contains some content from LTP and some part from BASID. The first part of the session (approximately 30–40 min) is focused on LTP and the second part (approximately 40–50 min) is dedicated to parents' mental health using BASID chapters. Three stories (Mr Rasheed, Ms Nasreen Bibi and Mr Muhammad Sabir) are referred to throughout the different sections of BASID such as how Muhamad Sabir prepared a list of graded exposure (session 5), how Muhammad Sabir identified his problems in problem-solving session (session 8), Nasreen Bibi's thought diary and thought challenging in session 9 and 10, Rasheed's example in session 12 on staying healthy (Table 1).

**Table 1.** LTP Plus TF CBT Intervention schedules

Session number	LTP	TF-CBT
1	Orientation to the intervention programme, Introduction to group rules, structure of sessions, frequency and timing of sessions etc.	
2	Introduction of the LTP Calendar; Overview of 5 domains of development – Sense of self, Physical Development, Relationships, Understanding, and Communication	Introduction of the fundamental principles of CBT
3	The younger 3 years old: Things that a younger 3 years old child shall do, role of parents (e.g. staying nearby the child when he/she tries new things, sharing books with children, story-telling etc), offering opportunities to learn (climbing, jumping, matching games etc)	Psychoeducation: What is trauma, symptoms of PTSD, vulnerable groups through three local culturally relevant stories. Relaxation exercise
4	The Older 3 years old: Things an older than 3 years old child shall do with pictorial illustrations. Helping parents to learn how to provide stimulation to the child such as make-believe plays, playing outside, learning how to take turns etc.	Ways to deal with fear, fight and flight responses. Problems related to sleep. Sleep hygiene
5	The younger 4 years old: Things a younger 4 years old child shall do. Helping parents how to teach their child different ways to express their feelings, settings routine of a child, providing them information about different body parts etc.	Ways to deal with Fear – Introduction to the graded exposure, preparing fear hierarchy (list of feared objects, activities and situations), ranking difficulty. An example from manual to be used. Encouraging to start with mildly difficult exposure
6	The younger 4 years old: Helping parents to provide stimulation for child development such as sense of self (offering them choices), physical development (drawing), relationship (inviting friends to play with child), parents engaging child in a play.	Ways to deal with Fear – Graded exposure Introducing coping strategies (deep breathing, counting backward, reciting verses from the Holy Quran, etc)
7	The older 4 years old: Things that an older 4 years old child shall do. Educating parents to provide time and space to the child to play. Examples of some easy play activities, helping parents in ensuring safety of the child.	Psychoeducation of vicious Activity scheduling
8	The older 4 years old: Helping parents to provide stimulation for child development such as sense of self (importance of answering child's questions in an honest way), physical development (outdoor play), relationship (helping child to recognise others' feelings and responding to others feelings), rhymes.	Problem solving – identifying problems and mutually exploring potential solutions using an example from the manual. Learning the technique of cost/benefit analysis
9	The younger 5 years old: Things a younger 5 years old child shall do (pictorial illustrations). Helping parents to understand importance of assigning simple jobs to the child, educating child about family's rules and how to offer child ways to explore their environment.	Helping participants to talk about thoughts and emotions. Physical manifestation of symptoms (e.g. of anxiety, fear) CBT cycle Thought keeping Diary
10	The younger 5 years old: Helping parents to provide stimulation for child development such as sense of self (appreciating child on completion of simple tasks), physical development (e.g. building something), solving problems on their own, looking at the calendar etc.	Thought challenging and practicing alternative healthy thoughts Thought Diary
11	The older 5 years old: Things and older 5 years old child shall do (pictorial illustrations). Helping parents to understand certain facts such as giving child an opportunity to say ‘no’, importance of giving opportunity to the child to experiment with different activities, listening to the child's stories attentively etc.	Communication skills Anger Management
12	The younger 5 years old: Helping parents to provide stimulation for child development such as sense of self (encouraging child when he gets frustrated while doing complex activities), physical development (e.g. crafting), counting patterns, sharing jokes etc. Certificate Distribution	Tips to stay healthy later on

### Research assistant (RA) training and inter-rater reliability

Training sessions for assessment measures are conducted for the RAs in Pakistan by senior trained professionals (NH, ZZ, FN, NC). To ensure consistency, local principal investigators (PIs) will measure the inter-rater reliability throughout the study. Moreover, those who deliver the intervention will attend a 3-day training on LTP Plus TF CBT by master trainers. These training sessions will involve a structured presentation on each session from the manual followed by a role play and discussion. These interventionists will be regularly supervised by the master trainers (AQ, ZZ, TK). These supervision sessions will be held on a fortnightly basis and include role play, case presentations and discussions.

For assessment of fidelity, we will use the participant observation method that we have already used in our published cluster RCT (Husain *et al*., 2021). On each study site (Peshawar and Karachi) two senior raters expert in LTP plus TF CBT will attend the sessions as delegates. Raters will develop and use a specific observation checklist which will be comprised of different domains from each age range of LTP and from each area of development from five areas identified in the LTP manual, as well as core components of TF CBT. The two raters will independently rate each session. Scores on the observation checklist will be reviewed for assessment of fidelity. In order to ensure consistency, the same two raters on each site will complete the fidelity assessments.

#### Treatment as usual (TAU)

The TAU group will receive routine treatment which may consist of support from general practitioners. The TAU also includes routine care provided by the CHWs (called Lady Health Workers in Pakistan). The CHWs are responsible for maternal, neonatal and child health in Pakistan and also take care of immunisation and family planning. Each CHW is responsible for approximately 150 households and they visit each household once a month (visiting 5–7 homes daily). They are also trained in interpersonal communication and community engagement.

All the participants in the TAU group will also receive detailed baseline and follow up assessment visits.

### Ethical considerations

Full ethical approval has been obtained from the ethics committee of the Institute of Professional Psychology (IPP), Bahria University Karachi, Pakistan. The research team will fully comply with the International Conference on Harmonisation Good Clinical Practice (ICH-GCP) guidelines.

#### Data protection and confidentiality

Unique identification (ID) numbers will be assigned to all trial participants and identifying information will only be accessible to the authorised researchers. Paper copies of assessment tools will all be stored in locked filing cabinets in a secured office. All anonymised data will be stored in an encrypted and password protected excel database. Interviews and focus group discussions will be digitally recorded with participants' consent to record. The interviews will then be transcribed. Recordings will be stored in a secure location and will be destroyed once the qualitative data is published. The quantitative data from the trial will be stored for 10 years in accordance to the Pakistan Institute of Living and Learning Data protection and Storage Policy.

### Adverse event reporting

All the adverse events, whether pertaining to the study or not, will be recorded in detail. In case of a serious adverse event, the PI will immediately notify the collaborating investigators and the ethics committee. Patient safety will be ensured by taking all the appropriate measures.

### Sample size

To have an effect size of 0.45, assuming 5% significance level, 80% power and 10% drop out (Husain *et al*., 2017b) during the study, we need to recruit 300 parents. The objective for which the larger sample size is needed relates to the intra-cluster correlation coefficient (ICC), which measures the degree of similarity in outcome amongst parents in the same cluster; for this, the key is the number of clusters (Rutterford *et al*., 2015). Moreover, groups (and hence clusters) of size around 10 is the optimal size for interventions such as LTP Plus TF-CBT (Biggs *et al*., 2020). We propose a minimum of 15 clusters in each of the LTP + TF-CBT and routine care arms, in order to be able to obtain a relatively precise estimate of the ICC and to check whether it might be greater in the LTP + TF-CBT and routine care arms as the former may have additional variation due to the delivery of the group-based therapy. We, therefore, expect to recruit around 300 parents (approximately 10 each from 15 LTP + TF-CBT and 15 TAU clusters) which will enable the estimation of recruitment and retention rates with sufficiently good precision to inform a full effectiveness trial.

## Assessment measures

### Assessment of feasibility and acceptability

The feasibility of the intervention will be assessed by;

- ascertaining whether the intervention is delivered, received and enacted as intended
- session completion rate monitored through session logs
- through qualitative feedback described below

Acceptability of the intervention will be assessed by;

- percentage dropouts due to non-acceptability
- rate of serious adverse events
- through qualitative feedback described below

**Demographic scale:** A study-specific demographic scale will be used to collect demographic information such as age, education, employment status, socio-economic status etc.

#### Primary outcome

The Clinician-Administered PTSD Scale-5 (CAPS-5) is a 30 item scale and will be used as a primary outcome measure as this is the gold standard structured interview for assessing PTSD, diagnostic status and symptom severity (Weathers *et al*., 2013). Questions target the onset and duration of symptoms, subjective distress, and impact of symptoms on social and occupational functioning. Research evidence with diverse cultures supports that CAPS is reliable and yields consistent scores across items (Weathers *et al*., 2001).

#### Secondary outcomes

Patient Health Questionnaire (PHQ-9) (Kroenke *et al*., 2001) to assess depression. The scale has been translated into Urdu and validated in Pakistan (Ahmad *et al*., 2018). The Urdu version has been used in previous trials in Pakistan (Husain *et al*., 2017b, 2020). The Cronbach's alpha for the PHQ-9 was reported to be 0.91 and the split-half reliability was 0.77 (Ahmad *et al*., 2018).

Generalised Anxiety Disorder Scale (GAD-7) (Spitzer *et al*., 2006) to assess anxiety. The scale has been translated into Urdu and validated in Pakistan (Ahmad *et al*., 2017). The Urdu version has been used in previous trials in Pakistan (Husain *et al*., 2017b, 2020). The Cronbach's alpha for the GAD-7 was reported to be 0.92 and split-half reliability was 0.82 (Ahmad *et al*., 2017).

Client Service Receipt Inventory (CSRI) (Beecham and Knapp, 2001) will be used to collect information about the use of other health services (including the informal sector faith healers/Imams). The CSRI has been used in a previous trial in Pakistan (Husain *et al*., 2014a).

Ages and Stages Questionnaire (ASQ-3) (Squires *et al*., 2009) to assess physical, social, emotional and cognitive development of children.

### Theory of Change (ToC)

The study team uses the Theory of Change approach as a standard framework for bringing about change. The community engagement process will be underpinned by the ToC (Mayne, 2020) causal model of planning, monitoring, evaluation and impact assessment to ensure that the marginalised voices are included in developing the vision. Barriers and challenges are identified through the lens of the end beneficiaries and short, medium and long term outcomes are developed and delivered so real change happens from the perspective of the target group. The ToC is closely embedded in community-based participatory research to engage the community for equity and to ensure that the process of Freirian collective reflection is in place. This reflection process aims to acknowledge the role of community knowledge and fit that can potentially impact the empowerment processes and consequently the power relations and greater equity outcomes within and outside partnerships (Wallerstein *et al*., 2020). The proposed composition of the stakeholder group for this project is truly reflective of the participatory approach by engaging parents with mental health difficulties, CHWs, health professionals, members of non-profit organisations and researchers. The study's ‘Patient and Public Involvement and Engagement (PPIE)’ group will be part of the stakeholder group. People with mental health difficulties make a vulnerable, stigmatised and marginalised group of population in our society (Vigo, 2016). This is particularly true for female survivors of mental health problems in Pakistan (Niaz, 2004). The involvement of the PPIE group in the ToC process throughout the project will ensure that the marginalised voices to be heard.

In practical terms at the planning stage, a workshop with stakeholders including persons with lived experience (the end beneficiaries) as the key stakeholder will come together to explore various elements of the ToC process by developing a shared vision and defining goals in the short, medium and longer term. This stakeholder engagement exercise will help to clarify roles and responsibilities as to who does what and how to achieve the vision. Assumptions will be articulated in the current context. Through our ToC process, stakeholders will identify the social and cultural factors and when the context changes, we will hold further stakeholder workshops to test our assumptions together in order to adapt and refine our model accordingly and with the ownership of all stakeholders. The theory of change will also enable us to show that trial results have been adopted in practice. What barriers and challenges were faced during the trial? What assumptions were made in defining the goal statements? The ToC approach will help mitigate some of the risks around stigma and refusal to participate as a representative group is involved at the inception of the trial. Our ToC pathway will give a detailed and direct understanding of the links between activities that lead to the desired goals. This understanding will lead to better evaluation and measure of progress and in the long term an understanding of impact both planned and unplanned.

### Statistical analysis plan

The study will be carried out in accordance with the CONSORT guidelines. All analyses will be based on the intention-to-treat sample and missing data will be imputed with multiple imputation procedures as implemented in Statistical Package for Social Sciences (SPSS) software (version 27). To assess the primary and secondary outcomes, a two-sided significance level of 0.05 will be employed to enable the detection of a difference between interventions in both directions. Demographics and baseline variables in the two study arms will be compared using various descriptive statistics of means, standard deviations, and proportions. For primary outcome analysis, a two-sample two-sided test of proportions will be used to compare the difference in the rate of PTSD (assessed using the CAPS-5) at 4-month post-randomisation between LTP Plus and TF-CBT and TAU groups. For the secondary analyses, we will compare the proportions similarly for binary outcomes and use tests for comparing means for the continuous outcome. Additionally, we shall use multivariable techniques in assessing the effect of various covariates including baseline measures on outcomes. If required appropriate transformation of continuous variables will be considered. ICC will be calculated to help future cluster-based research on this topic.

#### Qualitative component

In-depth digitally recorded interviews with participants and focus groups with key stakeholders will complement the quantitative data (Ritchie *et al*., 2013). This is to draw out complex issues that quantitative methods may overlook and to explore possible mechanisms of change in parents. Qualitative evaluation will include narrative experiences of health care providers, school teachers and other stakeholders (CHWs) in the project. Focus Groups will also be held separately with the participants (parents) and other family members, at the end of participation to explore perceived barriers and facilitators to the successful delivery of the intervention and optimise a framework for subsequent implementation at a national level. Separate topic guides will be developed to be used in focus group and interviews with participants and other stakeholders.

All the qualitative interviews and focus group discussions will be digitally recorded and transcribed verbatim. The transcripts will be translated into English. Initial 3–4 transcripts in English will be back translated into Urdu to ensure accuracy. Data will be coded using NVivo software. The process of analysis will be iterative as data collection will progress, using the principles of constant comparison, until category saturation will be achieved. Framework analysis will be conducted to analyse and interpret the qualitative data (Kiernan and Hill, 2018).

To maintain the credibility and trustworthiness of the data and subsequent findings, the researchers will be supervised by experienced researchers with expertise in qualitative methods (ZZ, TK). Sample of the transcripts will be discussed in regular meetings. Engagement in discussion and regular reviews by all researchers will help to ensure the fit of the data to the final analysis and help to minimise bias. Team members will agree the final theoretical framework and key themes and interpretations.

### Summary and implications

There are limited data available from RCTs to guide treatment choices for clinicians in Pakistan caring for patients, particularly parents with PTSD. As a result, there is a need to test low-cost, culturally sensitive, psychosocial interventions (LTP Plus) for parental PTSD in Pakistan. This study is particularly relevant to the achievement of the Sustainable Development Goals (SDGs) such as Health and Wellbeing (Goal 3), gender equality and empowering women (Goal 5), reducing inequalities (Goal 10) and reducing poverty (Goal 1) (Nations, 2016). The proposed study is an important extension of more than 20 years of work looking at the aetiology (Husain *et al*., 2007) and treatment of depression and anxiety in Pakistan (Husain *et al*., 2014b, 2017b, 2020), including a cultural adaptation of interventions (Naeem *et al*., 2015). In Pakistan, a parenting programme (Learning through Play-LTP) was tested for the first time through a cluster randomised study in a rural area of Rawalpindi, Pakistan (Rahman *et al*., 2009) Results indicated that the intervention group had a significantly higher increase in questionnaire scores of maternal knowledge about infant development than the control group at 3 months postpartum (with women in the intervention group answering correctly 4.3 [95% confidence interval (CI) 3.7–14.9, *p* < 0.001] more questions than the control group) but there were no difference in SRQ-20 (distress) scores between intervention and control groups (Rahman *et al*., 2009). A combination of a parenting programme (LTP) and Cognitive Behaviour Therapy was later tested with depressed mothers (Husain *et al*., 2017b, 2020). For both trials, at 3 months, there was a significant reduction in depression scores of mothers in the LTP Plus group compared to the control group. Since research supporting low-cost interventions is scarce, this trial would help build an understanding of the acceptability, feasibility and effectiveness of a low-cost parenting intervention and how it can help support parents experiencing trauma in Pakistan.

Supporting trauma survivor parents through stress reduction and management, parenting education and connecting them with others via group interventions can help them to positively impact the developmental trajectories of their children (Rosenblum *et al*., 2017). Such interventions are particularly important in Pakistan where women and children are the most vulnerable population because of fragile health systems and natural disasters (Jafar *et al*., 2013). A process evaluation nested in a pilot RCT from Pakistan highlighted that it is challenging for women to access facility-based mental health interventions (Rahman *et al*., 2016) therefore a community-based approach involving CHWs can be adopted to improve access to care (Chiumento *et al*., 2017).

## Trial status

This clinical trial was registered on 4th April 2018 (ClinicalTrials.gov, NCT03488290).

## References

[ref1] Ahmad S, Hussain S, Shah FS and Akhtar F (2017) Urdu translation and validation of GAD-7: a screening and rating tool for anxiety symptoms in primary health care. JPMA. The Journal of the Pakistan Medical Association 67, 1536–1540. 28955070

[ref2] Ahmad S, Hussain S, Akhtar F and Shah FS (2018) Urdu translation and validation of PHQ-9, a reliable identification, severity and treatment outcome tool for depression. JPMA. The Journal of the Pakistan Medical Association 68, 1166–1170. 30108380

[ref3] APA, American Psychological Association (2016) Trauma and Shock. Washington DC.: APA. Retrieved from http://www.apa.org/topics/trauma.

[ref4] Atwoli L, Stein DJ, Koenen KC and McLaughlin KA (2015) Epidemiology of posttraumatic stress disorder: prevalence, correlates and consequences. Current Opinion in Psychiatry 28, 307. 26001922 10.1097/YCO.0000000000000167 PMC4452282

[ref5] Beecham J and Knapp M (2001) Costing psychiatric interventions. Measuring Mental Health Needs 2, 200–224.

[ref6] Biggs K, Hind D, Gossage-Worrall R, Sprange K, White D, Wright J, Chatters R, Berry K, Papaioannou D and Bradburn M (2020) Challenges in the design, planning and implementation of trials evaluating group interventions. Trials 21, 116. 31996259 10.1186/s13063-019-3807-4 PMC6990578

[ref7] Centre, The Hincks-Dellcrest (2002) ‘The Learning Through Play Calendar: Training Manual’, *The Hincks-Dellcrest Centre, Toronto*, *Canada*.

[ref8] Chen JA, Olin CC, Stirman SW and Kaysen D (2017) The role of context in the implementation of trauma-focused treatments: effectiveness research and implementation in higher and lower-income settings. Current Opinion in Psychology 14, 61–66. 28713852 10.1016/j.copsyc.2016.11.007 PMC5507619

[ref9] Chiumento A, Hamdani SU, Khan MN, Dawson K, Bryant RA, Sijbrandij M, Nazir H, Akhtar P, Masood A and Wang D (2017) Evaluating effectiveness and cost-effectiveness of a group psychological intervention using cognitive behavioural strategies for women with common mental disorders in conflict-affected rural Pakistan: study protocol for a randomised controlled trial. Trials 18, 1–12. 28441974 10.1186/s13063-017-1905-8 PMC5405533

[ref10] Creech SK and Misca G (2017) Parenting with PTSD: a review of research on the influence of PTSD on parent-child functioning in military and veteran families. Frontiers in Psychology 8, 1101. 28713306 10.3389/fpsyg.2017.01101 PMC5491843

[ref11] Cross D, Vance LA, Kim YJ, Ruchard AL, Fox N, Jovanovic T and Bradley B (2018) Trauma exposure, PTSD, and parenting in a community sample of low-income, predominantly African American mothers and children. Psychological Trauma: Theory, Research, Practice, Policy 10, 327. 28481561 10.1037/tra0000264 PMC5677577

[ref12] Dubow EF, Huesmann LR, Boxer P, Landau S, Dvir S, Shikaki K and Ginges J (2012) Exposure to political conflict and violence and posttraumatic stress in Middle East youth: protective factors. Journal of Clinical Child Adolescent Psychology 41, 402–416. 22594697 10.1080/15374416.2012.684274 PMC3387283

[ref13] Ehlers A, Clark DM, Hackmann A, McManus F and Fennell M (2005) Cognitive therapy for post-traumatic stress disorder: development and evaluation. Behaviour Research Therapy 43, 413–431. 15701354 10.1016/j.brat.2004.03.006

[ref14] El-Khani A, Cartwright K, Redmond A and Calam R (2016) Daily bread: a novel vehicle for dissemination and evaluation of psychological first aid for families exposed to armed conflict in Syria. Global Mental Health 3, 1–7. 10.1017/gmh.2016.9 PMC5314739 28596884

[ref15] Ennis N, Shorer S, Shoval-Zuckerman Y, Freedman S, Monson CM and Dekel R (2020) Treating posttraumatic stress disorder across cultures: a systematic review of cultural adaptations of trauma-focused cognitive behavioral therapies. Journal of Clinical Psychology 76, 587–611. 31851380 10.1002/jclp.22909

[ref16] Foy DW, Eriksson CB and Trice GA (2001) Introduction to group interventions for trauma survivors. Group Dynamics: Theory, Research, and Practice 5, 246.

[ref17] Harrison D, Albanese P and Berman R (2014) Parent-adolescent relationships in military families affected by post-traumatic stress disorder. Canadian Social Work Review/Revue Canadienne de Service Social 31, 85–107.

[ref18] Husain N, Chaudhry IB, Afridi MA, Tomenson B and Creed F (2007) Life stress and depression in a tribal area of Pakistan. The British Journal of Psychiatry 190, 36–41. 17197654 10.1192/bjp.bp.106.022913

[ref19] Husain N, Afsar S, Ara J, Fayyaz H, Ur Rahman R, Tomenson B, Hamirani M, Chaudhry N, Fatima B and Husain M (2014a) Brief psychological intervention after self-harm: randomised controlled trial from Pakistan. The British Journal of Psychiatry 204, 462–470. 24676964 10.1192/bjp.bp.113.138370

[ref20] Husain N, Chaudhry N, Fatima B, Husain M, Amin R, Chaudhry IB, Ur Rahman R, Tomenson B, Jafri F and Naeem F (2014b) Antidepressant and group psychosocial treatment for depression: a rater blind exploratory RCT from a low-income country. Behavioural Cognitive Psychotherapy 42, 693. 23867053 10.1017/S1352465813000441

[ref21] Husain N, Chaudhry N, Furber C, Fayyaz H, Kiran T, Lunat F, Ur Rahman R, Farhan S and Fatima B (2017a) Group psychological intervention for maternal depression: a nested qualitative study from Karachi, Pakistan. World Journal of Psychiatry 7, 98. 28713687 10.5498/wjp.v7.i2.98 PMC5491481

[ref22] Husain N, Zulqernain F, Carter L-A, Chaudhry IB, Fatima B, Kiran T, Chaudhry N, Naeem S, Jafri F and Lunat F (2017b) Treatment of maternal depression in urban slums of Karachi, Pakistan: a randomized controlled trial (RCT) of an integrated maternal psychological and early child development intervention. Asian Journal of Psychiatry 29, 63–70. 29061430 10.1016/j.ajp.2017.03.010

[ref23] Husain N, Kiran T, Shah S, Rahman A, Saeed Q, Naeem S, Bassett P, Husain M, Ul Haq S and Jaffery F (2020) Efficacy of learning through play plus intervention to reduce maternal depression in women with malnourished children: a randomized controlled trial from Pakistan. Journal of Affective Disorders 278, 78–84. 32956964 10.1016/j.jad.2020.09.001

[ref24] Husain N, Kiran T, Fatima B, Chaudhry IB, Husain M, Shah S, Bassett P, Cohen N, Jafri F and Naeem S (2021) An integrated parenting intervention for maternal depression and child development in a low-resource setting: cluster randomized controlled trial. Depression and Anxiety 38, 925–939. 34010505 10.1002/da.23169

[ref25] Jafar TH, Haaland BA, Rahman A, Razzak JA, Bilger M, Naghavi M, Mokdad AH and Hyder AA (2013) Non-communicable diseases and injuries in Pakistan: strategic priorities. The Lancet 381, 2281–2290. 10.1016/S0140-6736(13)60646-7 23684257

[ref26] Khalily MT (2011) Mental health problems in Pakistani society as a consequence of violence and trauma: a case for better integration of care. International Journal of Integrated Care 11, 1–7. 10.5334/ijic.662 PMC3225239 22128277

[ref27] Khan F, Amatya B, Gosney J, Rathore FA and Burkle Jr FM (2015) Medical rehabilitation in natural disasters: a review. Archives of Physical Medicine Rehabilitation 96, 1709–1727. 25701639 10.1016/j.apmr.2015.02.007

[ref28] Kiernan MD and Hill M (2018) Framework analysis: a whole paradigm approach. Qualitative Research Journal 18, 248–261.

[ref29] Kroenke K, Spitzer RL and Williams JB (2001) The PHQ-9: validity of a brief depression severity measure. Journal of General Internal Medicine 16, 606–613. 11556941 10.1046/j.1525-1497.2001.016009606.x PMC1495268

[ref30] Latif M, Husain MI, Gul M, Naz S, Irfan M, Aslam M, Awan F, Sharif A, Rathod S and Farooq S (2020) Culturally adapted trauma-focused CBT-based guided self-help (CatCBT GSH) for female victims of domestic violence in Pakistan: feasibility randomized controlled trial. Behavioural Cognitive Psychotherapy 49, 1–12. 32993831 10.1017/S1352465820000685

[ref31] Mayne J (2020) Sustainability analysis of intervention benefits: a theory of change approach. Canadian Journal of Program Evaluation 35, 204–2021.

[ref32] Mihelic M, Morawska A and Filus A (2017) Effects of early parenting interventions on parents and infants: a meta-analytic review. Journal of Child Family Studies 26, 1507–1526.

[ref33] Montgomery E, Just-Østergaard E and Jervelund SS (2019) Transmitting trauma: a systematic review of the risk of child abuse perpetrated by parents exposed to traumatic events. International Journal of Public Health 64, 241–251. 30506365 10.1007/s00038-018-1185-4

[ref34] Naeem F, Taj R, Khan A and Ayub M (2012) Can watching traumatic events on TV cause PTSD symptoms? Evidence from Pakistan. Acta Psychiatrica Scandinavica 126, 79. 22582757 10.1111/j.1600-0447.2012.01876.x

[ref35] Naeem F, Phiri P, Munshi T, Rathod S, Ayub M, Gobbi M and Kingdon D (2015) Using cognitive behaviour therapy with south Asian Muslims: findings from the culturally sensitive CBT project. International Review of Psychiatry 27, 233–246. 26211879 10.3109/09540261.2015.1067598

[ref36] Nations, United, UN (2016) The Sustainable Development Goals 2016. USA: eSocialSciences.

[ref37] Niaz U (2004) Women's mental health in Pakistan. World Psychiatry 3, 60. 16633458 PMC1414670

[ref38] Olema DK, Catani C, Ertl V, Saile R and Neuner F (2014) The hidden effects of child maltreatment in a war region: correlates of psychopathology in two generations living in Northern Uganda. Journal of Traumatic Stress 27, 35–41. 24478246 10.1002/jts.21892

[ref39] Palosaari E, Punamäki R-L, Qouta S and Diab M (2013) Intergenerational effects of war trauma among Palestinian families mediated via psychological maltreatment. Child Abuse Neglect 37, 955–968. 23768956 10.1016/j.chiabu.2013.04.006

[ref40] Rahman A, Iqbal Z, Roberts C and Husain N (2009) Cluster randomized trial of a parent-based intervention to support early development of children in a low-income country. Child: Care, health development 35, 56–62. 18991970 10.1111/j.1365-2214.2008.00897.x

[ref41] Rahman A, Riaz N, Dawson KS, Hamdani SU, Chiumento A, Sijbrandij M, Minhas F, Bryant RA, Saeed K and van Ommeren M (2016) Problem Management Plus (PM+): pilot trial of a WHO transdiagnostic psychological intervention in conflict-affected Pakistan. World Psychiatry 15, 182. 27265713 10.1002/wps.20312 PMC4911784

[ref42] Ritchie J, Lewis J, Nicholls CM and Ormston R (2013) Qualitative Research Practice: A Guide for Social Science Students and Researchers. California, US: Sage.

[ref43] Rosenblum KL, Muzik M, Morelen DM, Alfafara EA, Miller NM, Waddell RM, Schuster MM and Ribaudo J (2017) A community-based randomized controlled trial of mom power parenting intervention for mothers with interpersonal trauma histories and their young children. Archives of Women's Mental Health 20, 673–686. 10.1007/s00737-017-0734-9 PMC5709180 28647759

[ref44] Rutterford C, Copas A and Eldridge S (2015) Methods for sample size determination in cluster randomized trials. International Journal of Epidemiology 44, 1051–1067. 26174515 10.1093/ije/dyv113 PMC4521133

[ref45] Song SJ, Tol W and De Jong J (2014) Indero: intergenerational trauma and resilience between Burundian former child soldiers and their children. Family Process 53, 239–251. 24635407 10.1111/famp.12071

[ref46] Spitzer RL, Kroenke K, Williams JB and Löwe B (2006) A brief measure for assessing generalized anxiety disorder: the GAD-7. Archives of Internal Medicine 166, 1092–1097. 16717171 10.1001/archinte.166.10.1092

[ref47] Squires J, Bricker DD and Twombly E (2009) Ages & Stages Questionnaires. Baltimore, MD, USA: Paul H. Brookes.

[ref48] Stein DJ, Karam EG, Shahly V, Hill ED, King A, Petukhova M, Atwoli L, Bromet EJ, Florescu S and Haro JM (2016) Post-traumatic stress disorder associated with life-threatening motor vehicle collisions in the WHO World Mental Health Surveys. BMC psychiatry 16, 1–14. 27449995 10.1186/s12888-016-0957-8 PMC4957291

[ref49] Sundin EC and Horowitz MJ (2002) Impact of event scale: psychometric properties. The British Journal of Psychiatry 180, 205–209. 11872511 10.1192/bjp.180.3.205

[ref50] Timshel I, Montgomery E and Dalgaard NT (2017) A systematic review of risk and protective factors associated with family-related violence in refugee families. Child Abuse Neglect 70, 315–330. 28683372 10.1016/j.chiabu.2017.06.023

[ref51] Vigo D (2016) The health crisis of mental health stigma. Lancet (London, England) 3, 171–178. 10.1016/S0140-6736(16)00687-5 27025171

[ref52] Wallerstein N, Oetzel JG, Sanchez-Youngman S, Boursaw B, Dickson E, Kastelic S, Koegel P, Lucero JE, Magarati M and Ortiz K (2020) Engage for equity: a long-term study of community-based participatory research and community-engaged research practices and outcomes. Health Education & Behavior 47, 380–390. 32437293 10.1177/1090198119897075 PMC8093095

[ref53] Watkins LE, Sprang KR and Rothbaum BO (2018) Treating PTSD: a review of evidence-based psychotherapy interventions. Frontiers in Behavioral Neuroscience 12, 258. 30450043 10.3389/fnbeh.2018.00258 PMC6224348

[ref54] Weathers FW, Keane TM and Davidson JR (2001) Clinician-administered PTSD scale: a review of the first ten years of research. Depression and Anxiety 13, 132–156. 11387733 10.1002/da.1029

[ref55] Weathers FW, Blake DD, Schnurr PP, Kaloupek DG, Marx BP and Keane TM (2013) The Clinician-Administered PTSD Scale for DSM-5 (CAPS-5), *Interview available from the National Center for PTSD at* www.ptsd.va.gov.

[ref56] Winston R and Chicot R (2016) The importance of early bonding on the long-term mental health and resilience of children. London Journal of Primary Care 8, 12–14. 10.1080/17571472.2015.1133012 PMC5330336 28250823

